# Anti-NMDA receptor encephalitis presenting as an acute psychotic episode misdiagnosed as dissociative disorder: a case report

**DOI:** 10.1186/s40981-016-0048-3

**Published:** 2016-09-01

**Authors:** Yuichiro Shimoyama, Osamu Umegaki, Tomoyuki Agui, Noriko Kadono, Toshiaki Minami

**Affiliations:** 1 Department of Anesthesiology, Osaka Medical College, 2-7 Daigaku-machi, Takatsuki, Osaka 569-8686 Japan; 2 Department of Surgery, Osaka Medical College, Takatsuki, Japan

**Keywords:** Limbic encephalitis, *N*-Methyl-d-aspartate (NMDA) receptor encephalitis, Paraneoplastic, Misdiagnosis

## Abstract

**Background:**

In 2005, “anti-*N*-methyl-d-aspartate (NMDA) receptor encephalitis,” a syndrome with prominent psychiatric symptoms, memory loss, decrease in level of consciousness, and central hypoventilation, was described in young women with ovarian teratomas and antibodies against an antigen highly expressed in the hippocampus. This report highlights the growing need for increased awareness among psychiatrists and other relevant medical professionals about this under-diagnosed disorder, which should be considered in differential diagnoses.

**Case presentation:**

A 19-year-old female with no psychiatric history presented to a district general hospital with acute psychosis, emotional lability, memory deficit, fluctuating behavioral changes such as wandering and babbling, and seizure. She was admitted to the hospital with a provisional diagnosis of dissociative disorder. Soon after admission, she developed aspiration pneumonia and was intubated for mechanical ventilation. She was transferred to our hospital for further assessment and admitted to the intensive care unit for ventilation. Laboratory test results were unremarkable, but her EEG showed non-specific slowing with no epileptiform activity, and brain computed tomography (CT) and MRI also showed no remarkable findings. Cerebrospinal fluid (CSF) analysis showed an elevated white blood cell count (15 cells/hpf; 70 % lymphocytes), and blood serum and CSF samples tested positive for NMDA receptor antibodies. Abdominal contrast-enhanced CT revealed an ovarian teratoma, which was subsequently removed laparoscopically. Postoperative immunotherapy (steroids, intravenous immunoglobulin, and plasmapheresis) led to gradual improvement. On day 25 of hospitalization, neuropsychological assessment demonstrated that overall, she had returned to her premorbid level of functioning. Her condition substantially improved over several months of cognitive rehabilitation, and she was eventually discharged on day 75.

**Conclusions:**

Anti-NMDA receptor encephalitis, a form of autoimmune encephalitis, is commonly associated with tumors and often misdiagnosed. Diagnosis can be confirmed by detecting NMDA receptor antibodies in the patient’s serum or CSF. Management can be achieved with immunosuppressive therapy and tumor resection.

## Background

In 2005, “anti-*N*-methyl-d-aspartate (NMDA) receptor encephalitis,” a syndrome with prominent psychiatric symptoms, memory loss, decrease in level of consciousness, and central hypoventilation, was described in young women with ovarian teratomas and antibodies against an antigen highly expressed in the hippocampus. When severe, this disorder can be life-threatening, requiring intensive care treatment [1]. Here, we present the case of a 19-year-old female with anti-NMDA receptor encephalitis who was first diagnosed with dissociative disorder. The present case is noteworthy because of the dramatic and rapid recovery from surgery she made before returning to her premorbid level of functioning. Anti-NMDA receptor encephalitis is often misdiagnosed as viral encephalitis, neuroleptic malignant syndrome, or acute psychotic disorder, but treatment is specific and delays are associated with poor outcomes. As such, this report highlights the growing need for increased awareness among psychiatrists and other relevant medical professionals about this under-diagnosed disorder, which should be considered in differential diagnoses.

## Case presentation

**Fig. 1:**
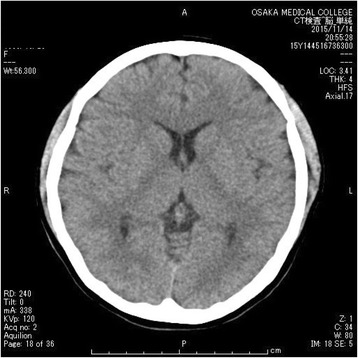
Head CT image with no remarkable findings

**Fig. 2:**
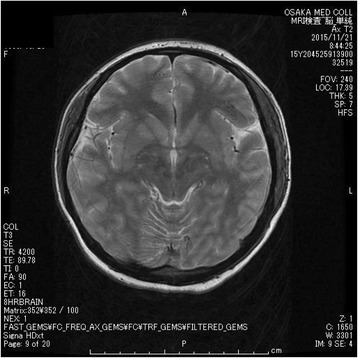
Brain MRI image with no remarkable findings

**Fig. 3:**
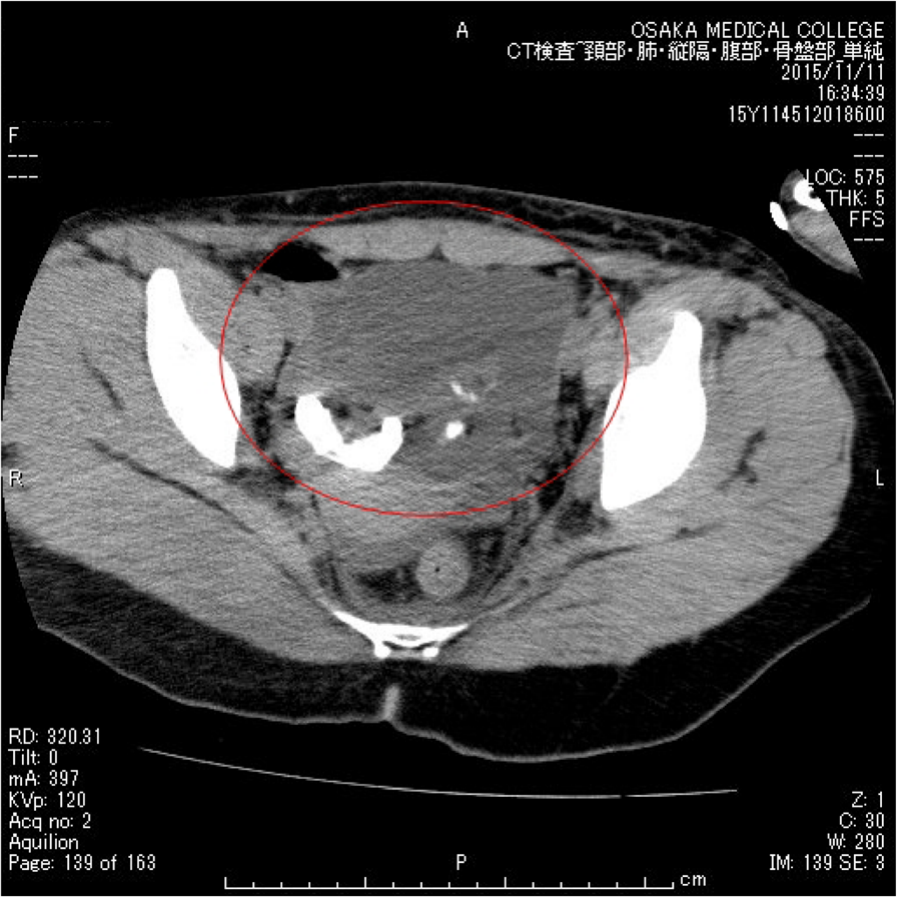
Abdominal CT image showing a right cystic adnexal mass with an internal focus of fat and high-attenuation material, suggesting an ovarian teratoma (*red circle*)

A 19-year-old female with no psychiatric history presented to a district general hospital with a history of acute psychosis, emotionally lability, memory deficit, fluctuating behavioral changes such as wandering and babbling, and seizure. Neurological examination did not reveal any focal signs, and routine laboratory workup was normal. She was admitted to the hospital with a provisional diagnosis of dissociative disorder. Soon after admission, she developed aspiration pneumonia and was intubated for mechanical ventilation. At the time, repeated seizures occurred every 10 min. Although ancillary tests including magnetic resonance imaging (MRI), electroencephalogram (EEG), and cerebrospinal fluid (CSF) analysis were unremarkable, the presentation of acute psychosis in combination with recurrent seizures and a relentless course suggested autoimmune encephalitis. She was transferred to our hospital for further assessment and admitted to the intensive care unit (ICU) for ventilation, where sedation was performed with propofol and dexmedetomidine. Her condition upon arrival was as follows: Glasgow Coma Scale score 1-T-1, respiratory rate 12 breaths/min on ventilation, pulse rate 80 beats/min, blood pressure 110/79 mmHg, and body temperature 36.6 °C. There were no notable laboratory test results, her EEG showed non-specific slowing with no epileptiform activity, CSF analysis showed an elevated white blood cell count (15 cells/hpf; 70 % lymphocytes), and brain computed tomography (CT) and MRI showed no remarkable findings (Figs. 1 and 2). Her blood serum and CSF samples collected at the time of arrival were sent to Dr. Dalmau’s laboratory for the measurement of anti-NMDA receptor antibody levels. The acute presentation of a severe psychotic state, with cognitive impairment, fluctuating behavioral changes, and recurrent seizures led to a clinical diagnosis of probable autoimmune encephalitis. However, as the possibility of viral encephalitis remained, intravenous acyclovir was initiated and continued until the results of polymerase chain reaction of CSF samples were found negative 1 week later. Given the suspicion of anti-NMDA receptor encephalitis, abdominal contrast-enhanced CT (Fig. 3) was performed, and a cystic lesion with calcification in the pelvis was identified. The lesion was determined to be an ovarian teratoma and was subsequently removed laparoscopically. Postoperatively, she underwent a total of seven plasmapheresis treatments (on days 2, 5, 7, 9, 14, 16, and 19), as well as betamethasone (first at 32 mg and gradually decreased) and IVIg (0.4 g/kg/day) for 5 days, which led to gradual improvement. On postoperative day 12, she was extubated, and the administration of propofol and dexmedetomidine was stopped. Soon after, the patient experienced a generalized tonic-clonic seizure. She was treated with levetiracetam 500 mg/day and did not experience a second generalized tonic-clonic seizure. On day 25 of hospitalization, blood serum and CSF samples were found positive for anti-NMDA receptor antibodies, leading to a confirmed diagnosis of anti-NMDA receptor encephalitis. Neuropsychological assessment performed at that time demonstrated that overall, she had returned to her premorbid level of functioning. She showed profound improvement over several months of cognitive rehabilitation and was eventually discharged on day 75. After discharge, she was referred to a cognitive rehabilitation day service facility. Since then, she has not experienced any relapse in generalized tonic-clonic seizures or other symptoms. The patient returned to school 2 months after discharge.

### Discussion

Anti-NMDA receptor encephalitis is a form of autoimmune encephalitis first reported in 2007 by Dalmau et al. of the University of Pennsylvania [2]. In October 2008, clinical data from 100 cases, gathered from around the world, were published in *Lancet Neurology* [3]. This type of encephalitis often occurs in young women at a median age of 23 years, with a male-to-female ratio of 9:91 [3]. Patients with this disease exhibit psychiatric symptoms, involuntary movement of the face and limbs, disturbance of consciousness, and central hypoventilation. In the early phase, patients develop many different psychiatric symptoms, but they follow a very similar course—gradually improving after progressing through common cold symptoms, a period of psychiatric symptoms, an immobile period, and a hyperactive period [4]. Some cases of anti-NMDA receptor antibodies identified in patients with purely psychiatric disorders have also been reported [5, 6]. The complication rate for teratomas is reported to be 59, and 95 % of all teratomas are ovarian teratomas; additionally, mediastinal teratoma, orchioncus, small cell lung carcinoma, and other tumors have been reported [7]. Definitive diagnosis is possible when anti-NMDA receptor antibodies are detected in the CSF and blood serum.

We present a case of successful treatment of paraneoplastic anti-NMDA receptor encephalitis. In this case, a 19-year-old woman was admitted following an abrupt-onset episode of emotional lability, memory deficit, fluctuating behavioral changes such as wandering and babbling, and seizure. Although routine physical and laboratory tests were normal, the appearance of fluctuations in mental status combined with repeated generalized seizures, results of spinal fluid examination with an elevated number of lymphoid cells, and slow activity in EEG initially led to a diagnosis of suspected limbic encephalitis. The current management guidelines for this disorder advocate early detection and removal of the teratoma [1]. In our case, the discovery of a teratoma by abdominal CT suggested a diagnosis of anti-NMDA receptor encephalitis, and the teratoma was urgently removed even before the results of anti-NMDA receptor antibodies in CSF were obtained.

In general, the majority of patients present with a combination of behavioral, cognitive, and motor symptoms, as well as speech disorder, seizures, and decreased level of consciousness, with psychiatric symptoms often predominating in the early phase. Such a presentation often leads patients to first seek psychiatric evaluation and treatment, leading to a crucial delay in diagnosis and immunotherapy [8]. Moreover, a recent large cohort-based study revealed that 4 % of patients presented with isolated psychiatric episodes (pure psychiatric symptoms without neurological involvement) [9]. In this cohort, brain MRI, EEG, and CSF studies were abnormal in 33, 90, and 79 % of patients, respectively. Moreover, results of these ancillary tests in patients with isolated psychiatric episodes were similar to the cohort population at large. As early recognition of these episodes and initiation of appropriate therapy was shown to be an important prognostic factor, the authors recommended that, in patients with new-onset psychosis, history of encephalitis, subtle neurological symptoms, and abnormal, albeit non-specific, CSF, EEG, or MRI findings, prompt screening should be performed for NMDA receptor antibodies and ovarian teratomas when applicable [9]. In the present case, although the patient was first diagnosed with dissociative disorder at a district general hospital, prompt screening for NMDA receptor antibodies, early detection and removal of the ovarian teratoma, and immunotherapy following surgery were adequately performed at our hospital after transfer.

Recent data suggest that over half of patients respond to first-line immunotherapy (steroids, intravenous immunoglobulin, and plasmapheresis, alone or in combination) within 4 weeks. Moreover, second-line treatment (rituximab and/or cyclophosphamide) is usually effective when first-line therapies fail. When relevant, rapid removal of an associated neoplasm is warranted and possibly reduces the future risk of relapse, which may exceed 10 % during the first 2 years. Despite optimal treatment, the recovery process can continue for over 18 months [1]. A prolonged hospital stay is to be expected with a reported median hospitalization period of 2.5 months (range, 2 to 14 months) [4]. These interventions resulted in marked improvements over the course of several months in this case. Notably, neuropsychological assessment performed on day 25 of hospitalization demonstrated that overall, she had returned to her premorbid level of functioning. The present case represents a relatively short time to recovery relative to that of previous reports [2].

This patient underwent general anesthesia for her surgery. In general, preoperative measures to prevent deep vein thrombosis are required because the duration of sedation under mechanical ventilation can sometimes be quite long in these patients. In addition, during surgery, the use of ketamine and nitrous oxide should be avoided, as they inhibit NMDA receptors (NMDAR) [10] and volatile anesthetics exhibit a wide range of NMDAR inhibitory potencies and immobilizing activities. By contrast, propofol is less likely to produce anesthetic effects through an NMDAR-dependent mechanism. This is because propofol anesthetizes via enhancing GABA-ergic transmission. Therefore, propofol may be a more appropriate anesthetic for such patients [10]. Postoperatively, many patients require mechanical ventilation to address respiratory depression and seizures.

In summary, the present case illustrates that, while anti-NMDA receptor encephalitis is an unfamiliar condition to many physicians, the inclusion of this disorder in differential diagnoses is critical, as prompt initiation of immunotherapy and tumor removal, if appropriate, could dramatically improve outcomes.

## Conclusions

Anti-NMDA receptor encephalitis is an often misdiagnosed form of autoimmune encephalitis commonly associated with tumors. Detection of NMDA receptor antibodies in the patient’s serum or CSF is necessary for a confirmed diagnosis, and management is achieved with early immunotherapy and tumor removal.

## Abbreviations

NMDA: *N*-Methyl-d-aspartate
MRI: Magnetic resonance imaging
CSF: Cerebrospinal fluid
ICU: Intensive care unit
CT: Computed tomography
